# Ecological successions throughout the desiccation of Tirez lagoon (Spain) as an astrobiological time-analog for wet-to-dry transitions on Mars

**DOI:** 10.1038/s41598-023-28327-3

**Published:** 2023-02-08

**Authors:** Alberto G. Fairén, Nuria Rodríguez, Laura Sánchez-García, Patricia Rojas, Esther R. Uceda, Daniel Carrizo, Ricardo Amils, José L. Sanz

**Affiliations:** 1 Centro de Astrobiología (CSIC-INTA), 28850 Torrejón de Ardoz, Spain; 2 Department of Astronomy, Cornell University, Ithaca, NY 14853 USA; 3 Centro de Biología Molecular Severo Ochoa (CSIC-UAM), Universidad Autónoma de Madrid, Cantoblanco, 28049 Madrid, Spain; 4 Departamento de Biología Molecular, Universidad Autónoma de Madrid, Cantoblanco, 28049 Madrid, Spain

**Keywords:** Ecology, Microbiology, Systems biology, Ecology, Environmental sciences, Planetary science, Astronomy and planetary science

## Abstract

Tirez was a small and seasonal endorheic athalassohaline lagoon that was located in central Spain. In recent years, the lagoon has totally dried out, offering for the first time the opportunity to analyze its desiccation process as a “time-analog” to similar events occurred in paleolakes with varying salinity during the wet-to-dry transition on early Mars. On the martian cratered highlands, an early period of water ponding within enclosed basins evolved to a complete desiccation of the lakes, leading to deposition of evaporitic sequences during the Noachian and into the Late Hesperian. As Tirez also underwent a process of desiccation, here we describe (i) the microbial ecology of Tirez when the lagoon was still active 20 years ago, with prokaryotes adapted to extreme saline conditions; (ii) the composition of the microbial community in the dried lake sediments today, in many case groups that thrive in sediments of extreme environments; and (iii) the molecular and isotopic analysis of the lipid biomarkers that can be recovered from the sediments today. We discuss the implications of these results to better understanding the ecology of possible Martian microbial communities during the wet-to-dry transition at the end of the Hesperian, and how they may inform about research strategies to search for possible biomarkers in Mars after all the water was lost.

## Introduction

The formation of widespread evaporite-bearing sediments on Mars has been extensively investigated by several spacecraft for decades^1,2^. These orbiter and rover investigations have revealed both water ponding within enclosed basins and groundwater activity, with enhanced evaporation processes and deposition of evaporitic sequences, all across the southern highlands during the Noachian and well into the Hesperian^3–5^ (ending 3.2 Ga). The diverse hypersaline aqueous environments from which the evaporites precipitated would have secured habitable conditions during a significant fraction of the early history of Mars^6–8^, as can be tested in analog environments on Earth today^9^. Previous studies conducted in hypersaline lagoons on Earth have been discussed in the context of Mars habitability^10^. Here we investigate another analog environment for early Mars, Tirez, a small and seasonal endorheic hypersaline lagoon that was located in western La Mancha, Spain, and which became totally desiccated in the recent years.

Tirez is located at 653 m altitude, N 39°32′12.12″ and W 03°21′33.47″. The Tirez lagoon was a small body of water, had a maximum extension of 0.8 km^2^ and a water column of 40 cm depth, within a poor drainage basin unrelated to any fluvial network, placed in a region with normally scarce rain and a high level of summer evaporation (Table 1, and see Supplementary Information for details). Since 2015, Tirez is completely dry. We closely witnessed and studied this ecological hardship during 25 years of continuous monitoring of Tirez, and used it as an opportunity to better understand the evolution of microbial communities subjected to evaporation and final desiccation in small lagoons.

**Table 1:** Physico-chemical parameters, anions and cations of the Tirez lagoon (April 2002).

	Water column	Sediment (0–2 cm)	Interstitial water^1^ (0–2 cm)
pH	9.0	7.5	7.7
Conductivity (mS/cm)	88	23.2	97.7
E_h_′ (mV)	115	− 132	n.s.
O_2_ dissolved (mg/l)	5.5	0.11	n.s.
Temperature (°C)	n.d.	22	n.s.
Hardness (as CaCO_3_)	32.5	n.d.	52.5
SO_4_^2−^	40.3	n.d.	44.8
Cl^−^	34.3	n.d.	27.4
NO_3_^−^	1.0	n.d.	0.34
Na^+^	13.9	n.d.	16.9
K^+^	1.0	n.d.	1.4
Mg^2+^	11.4	n.d.	15.2
Ca^2+^	0.5	n.d.	0.4

Concentrations are expressed as g L^−1^.

^1^Measurements were taken with interstitial water obtained by centrifugation of the sediments.

*n.d.* not determined, *n.s.* no sense.

The following physico-chemical parameters were measured in situ before the desiccation of the lagoon: temperature and conductivity (Orion 120), pH and redox potential (Orion 420), and dissolved oxygen (Symplair Sylaud Ins.). Sulfate and carbonate were also determined in situ with Hanna Instruments kits (Hanna Sulfate LR-HR H1-38001A for sulfate and Hardness HR H1-3812 for carbonate). Other anions and cations (Cl^−^, Na^+^, K^+^, Mg^2+^, Ca^2+^) were determined in the laboratory by elemental TXRF analysis and NO_3_^−^ by ionic chromatography.

**Figure 1:**
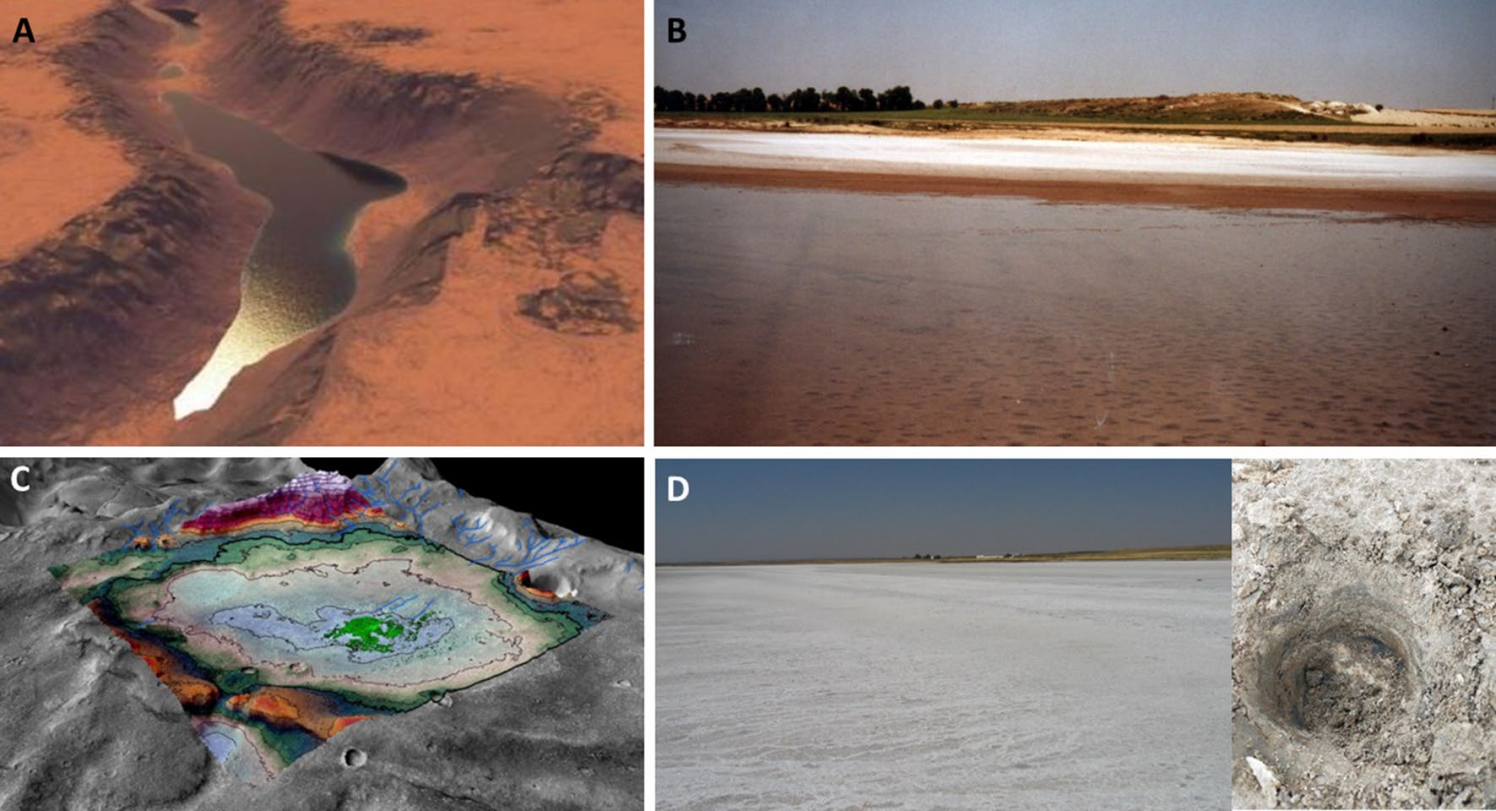
(**A**) Artistic impression showing ancient lakes ponded on Mars (credit: G. Di Achille, University of Colorado); (**B**) photograph of Tirez taken in 2002, when the lake was still active; (**C**) detection of evaporite salts in shallow basins in Meridiani (for interpretation, see Ref.^3^); (**D**) dry Tirez as it looks today, showing the evaporite salts, and an inset detailing the drilled sediments for this study.

It is expected that the conditions imposed by the desiccation of the lagoon may have modified the original prokaryotic microbiota, and the sediment today probably harbors microbial communities different from those observed in the lagoon and sediment before desiccation and, perhaps, different from those found in other lagoons subjected to less extreme (i.e., non-hypersaline and arid) conditions. With the aim of addressing this gap in our knowledge of ecological successions in the prokaryotic communities inhabiting athalassohaline small lagoons which have subsequently experienced desiccation, in this work we present for the first time a description of the changes in the microbial community of a desiccating athalassohaline endorreic lagoon, analyzing sediment samples from (i) 2002 when Tirez was covered by water, by the generation of a clone library, and (ii) 2021 when Tirez was completely dry, by massive sequencing and molecular and isotopic analysis of lipid biomarkers. We focus our analyses on prokaryotes because, if life ever existed on Mars, it most probably was prokaryotic-like, considering the time it took for eukaryotes to appear on Earth since prokaryotes did^11^. We use this pioneering long-run study to draft preliminary analogies to possible ephemeral lacustrine environments subjected to similar desiccation processes that could have occurred in several locations globally on Mars^12^, at the end of the Hesperian period (Fig. 1). This study introduces the concept of “astrobiological time-analog” (as opposed to the classic analogs^9^ presented as comparable locations in space, or “astrobiological site-analogs”) to possible sequential processes occurring in desiccating lakes on Mars billions of years ago.

## Results

### Microbial biodiversity in the active lake in 2002

#### Bacterial domain

192 clones were sequenced in 2002, which after the elimination of incomplete sequences and sequences belonging to the vector, were reduced to 125 complete sequences (closed to 1500 nucleotides). According to the BLAST analysis, these sequences could be classified into 11 different taxa (to the genus/species level), with the following similarities: Epsilonproteobacteria (74 clones distributed in three subgroups, affiliated to the genus *Malaciobacter*); Deltaproteobacteria (6 clones affiliated to *Desulfotignum* sp.); Bacillota (previously named Firmicutes), distributed between classes Bacilli (24 clones grouped in two taxonomic units related to genus *Virgibacillus* (previously named *Bacillus*), and Clostridia (10 clones affiliated to *Tissierella*); Spirochaetota (previously named Spirochetes) (7 clones), and 4 clones of uncertain adscription (Table 2).

**Table 2:** Phylogenetic affiliation of sequences obtained from clones from Tirez Lagoon in 2002.

OTU	Clons (%)	Phylogeny (genus)	Closer specie (% similarity)
Sal B35	28.8	*Malaciobacter*	*M. canalis* (98%)
Sal B7	27.2	*Malaciobacter*	*M. halophilus* (91)
SalB14	3.2	*Malaciobacter*	*M. canalis/M. marinus* (90)
SalB6	11.2	*Virgibacillus*	*V. halodenitrificans* (89%)
SalB20	8	*Virgibacillus*	*V. halodenitrificans* (93.8)
SalB34	4.8	*Tissierella/Gudongella*	T. sp./*G. oleilytica* (94.5)
SalB15	3.2	*Tissierella*	*Tissierella* sp. (98.5)
SalB38	4.8	*Desulfotignum*	*D. toluenicum* (96.6)
SalB9	3.2	Rikenellaceae/*Clostridium*	KJ955676/JQ724327 (94)
SalB63	5.6	Spirochaeta	Uncultured Spirochaetes (94.7)
SalA34/A36	100	*Methanohalophilus*	*M. portucalensis/M. mahii/M. halophilus* (99.3)

#### Archaeal domain

The amplified products were analyzed, prior to sequencing, according to their restriction profile with Sau3AI due to the low level of expected diversity. All the clones grouped in only two restriction patterns. The 16S rRNA sequences showed high homology to the genus *Methanohalophilus* only. Using specific assays, it was determined in 2002 laboratory enrichment cultures that the sediments had sulfate-reducing activity (measured as production of H_2_S), and methanogenic activity (measured as production of CH_4_), the most characteristic activities of anaerobic environments.

### Microbial biodiversity in the dried lake sediments in 2021

#### Bacterial domain

**Figure 2:**
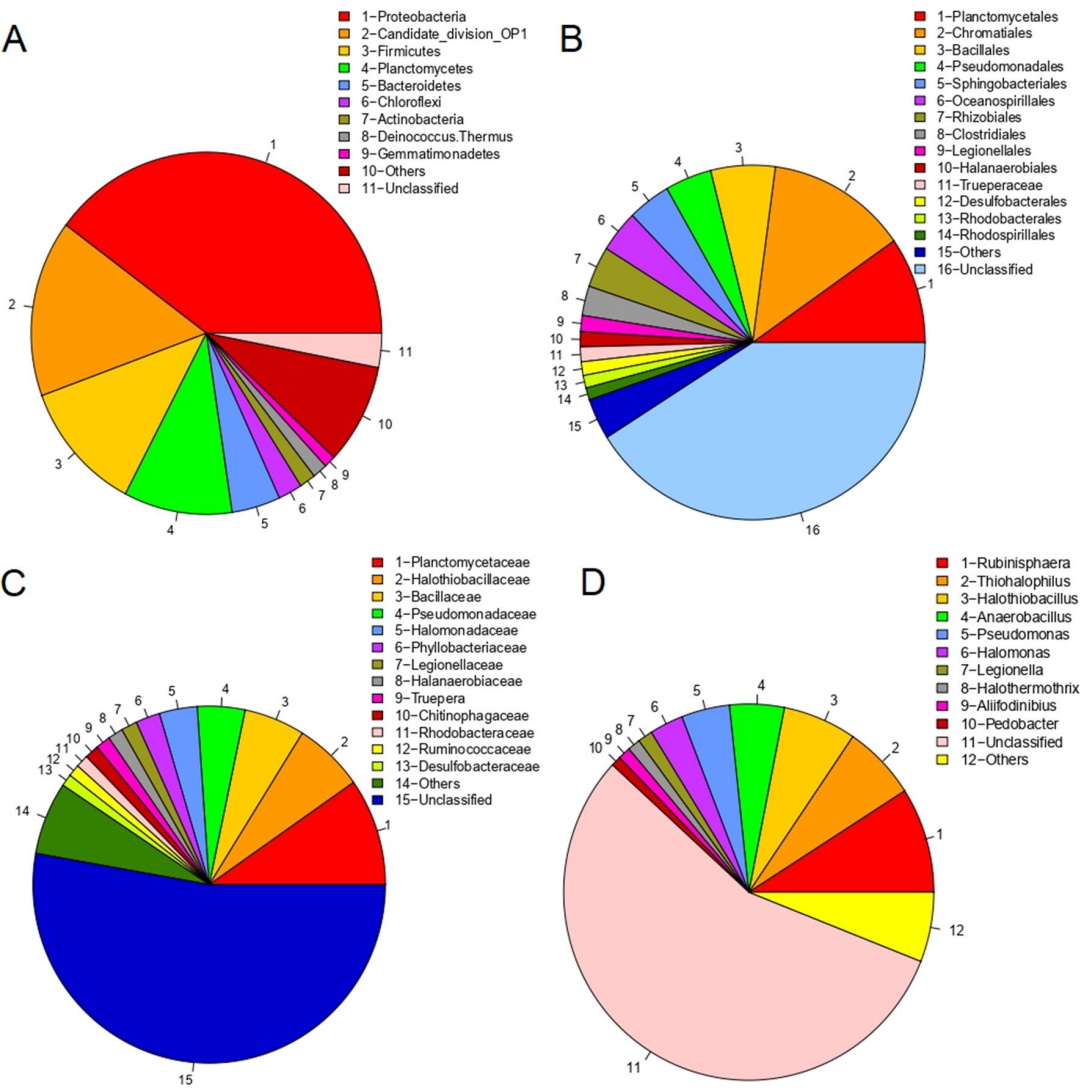
Pie charts of phylogenetic profiles for Bacteria Domain in Tirez sediments analyzed in 2021, at phylum (**A**), order (**B**), family (**C**) and genus (**D**) level. Taxa with a coverage lower than 1% of the total number of sequences have been grouped as “Others”. OTUs have been defined at specie level (97% similarity cutoff).

40% of the reliably assigned sequences at the phylum level were within Pseudomonadota (previously named Proteobacteria). This phylum, together with Bacillota, Planctomycetota (previously named Planctomycetes) and the Candidate division OP1 covered 77% of the total high-quality sequences (Fig. 2A). Pseudomonadota were mainly distributed among the orders Chromatiales (genera *Thiohalophilus* and *Halothiohalophilus*), Pseudomonadales (genus *Pseudomonas*), and Oceanospiralles (genus *Halomonas*). Bacillota were distributed between Bacillales and Clostridiales orders. Most of the Bacillales were related to the cluster Anaerobacillus/Halolactibacillus (previously named Bacillus). The Clostridiales were affiliated to the families Halanaerobiaceae, Ruminococcaceae or they were unclassified at family and genus levels. All Planctomycetota (previously named Planctomycetes) were within order Planctomycetales, genus *Rubinisphaera* (previously named^13^ *Plactomyces*) (Fig. 2B–D). 16% of the sequences were within Candidate division OP1 and could not be classified a higher taxonomic level.

The bacterial data (Table 3 and Fig. 2) show three clear results. First, the high values of the specific richness (Sobs), Shannon (H) and Chao 1 indexes show a high biodiversity. Although most of the reads were affiliated to 9 phyla, a further 19 phyla were also identified, albeit with less than 1% coverage. Similarly, high-quality sequences affiliate to 110 orders, 150 families, and 310 OTUs at the genus level. Furthermore, 86% of the OTUs (at species level) were raretons (contain only one or two sequences). The second result is that the Gini index evidences a high inequality. The Simpson index, close to zero, confirmed this statement. In addition, the coverage of the 10 major orders, families and genera reaches approximately 70% of the total sequences at that taxonomic level. And the third result is that a significant fraction of the sequences was within clusters with no isolated members (e.g*.,* Candidate division OP1, AKYG1722, or PAUC43f_marine_benthic_group) or unclassified at the considered taxa level. In other words, 41%, 53%, and 63% of the sequences could not be classified at the level of order, family and genus, respectively. The community of microorganisms that can be detected with high throughput methods such as direct DNA sequencing, but which phylogenetic identity still cannot be determined, has been recently named^14^ as “Dark Microbiome”.

**Table 3:** Coverage, diversity and evenness indices of the Tirez sediment in 2021.

	Bacteria	Archaea
Total reads (sequences)	90,934	110,464
High quality reads	73,382	108,939
OTUs (at 97%)	10,019	6429
Raretons	8603	5199
Average size (bp)	459	421
S_OBS_	10,019	6429
Chao1	33,842 ± 1692	19,941 ± 1127
Good’s coverage	0.8047	0.7324
Shannon (H)	6.87 ± 0.03	7.35 ± 0.03
Simpson	0.011 ± 0.001	0.005 ± 0.0002
Shannon evenness	0.7454	0.8383
Gini	0.9027	0.7892

#### Archaeal domain

The 9.7% of the high-quality sequences could only be affiliated to the Archaea domain. The remaining sequences were within the Euryarchaeota phylum, although only 73% could be assigned to the class level: Thermoplasmata (34.8%), Halobacteria (26.2%), Methanomicrobia (6.1%), Archaeglobi (5.9%) and Marine Benthic Group B (2.7%). All the Halobacteria were affiliated to Halobacteriaceae family, but only *Halobacterium* (15.6% of the Halobacteria) and *Natronomonas* (2.5 of the Halobacteria) could be to genus taxa; all the Methanomicrobia were within the unculture ST-12K10A group. The sequences retrieved from the sample affiliated to other classes: i.e., Thermoplasmata, Archaeglobi, and Marine_bentic-group_B, could not be classified at genus or family level. The most abundant OUT (Operational Taxonomic Unit) was KTK4A (13.2% of the total archaeal sequences), within Thermoplasmatales.

The analysis of the archaeal data (Table 3) shows two clear results. First, the ecological indexes show high diversity (Sobs, H, Chao) and lack of uniformity (Simpson, Shannon evenness). These indexes, together with the Gini index, the number of OTUs, and the percentage of raretons (81% of the OTUs at species level) indicate that archaea have less diversity than bacteria. And second, only 49%, 32% and 5% of the high-quality reads could be reliably assigned to order, family and genus level, respectively, and therefore are representatives of the “Dark Microbiome”^14^, as most of the sequences were unclassified or belonged to groups with no isolated members.

### Molecular and isotopic composition of lipid biomarkers in 2021

**Figure 3:**
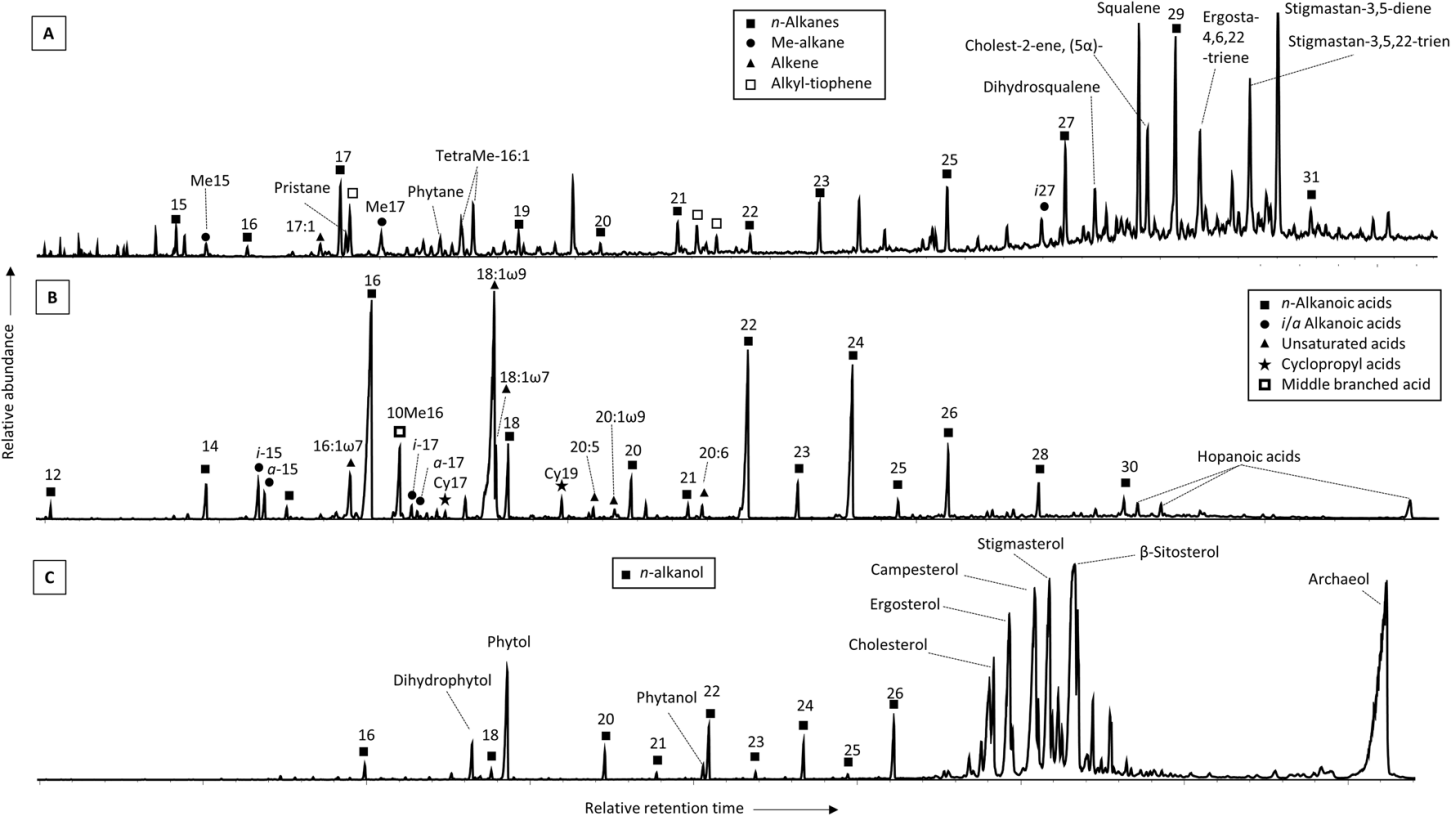
Mass chromatograms (represented as total ion current) of the three polarity fractions extracted from the dry sediments in the Tirez lagoon in 2021; (**A**) apolar fraction containing linear and saturated (*normal*) alkanes (*n*-alkanes), branched (methylated) and unsaturated (chains with double bonds) hydrocarbons, isoprenoids, and steranes; (**B**) acidic fraction with alkanoic acids of *normal*, branched, unsaturated, and cyclopropyl chains; and (**C**) polar fraction including *n*-alkanols, phytol and derivatives, archaeol and sterols.

The apolar fraction in the dried sediment sample from Tirez lagoon in 2021 was composed of a series of “normal” (i.e., linear and saturated chain) alkanes from *n*-C_14_ to *n*-C_29_, the alkene heptadecane (C_17:1_), some mid-chain monomethyl alkanes (7Me-C_15_ and 7Me-C_17_) and dimethyl octadecane (DiMe-C_18_), organosulfur compounds (alkyl-thiophens), abundant isoprenoids (mostly squalene, dihydrosqualene, tetrahydrosqualene, pristane and phytane), and some steranes (i.e., sterol degradation products) (Fig. 3A). In the acidic fraction, we identified a series of *n*-alkanoic acids from C_12:0_ to C_30:0_, dominated by *n*-C_16:0_, *n*-C_22:0_, and *n*-C_24:0_; some mono- (C_16:1[ω6]_, C_17:1_, C_18:1[ω9]_, C_18:1[ω7]_, and C_20:1[ω9]_) and poly- (C_18:2[ω6]_, C_20:5_, and C_20:6_) unsaturated acids; terminally branched (*iso*/*anteiso*) alkanoic acids from C_12_ to C_17_; cyclopropyl acids (Cy_17_ and Cy_19_); and a mid-chain monomethyl alkanoic acid (10MeC_16:0_) (Fig. 3B). The polar fraction was dominated by archaeol and a number of eukaryotic sterols (ergosterol, campesterol, stigmasterol, β-sitosterol, and cholesterol), and showed minority concentration of phytol, phytanol, and *n*-alkanols from C_12_ to C_28_ (Fig. 3C).

**Figure 4:**
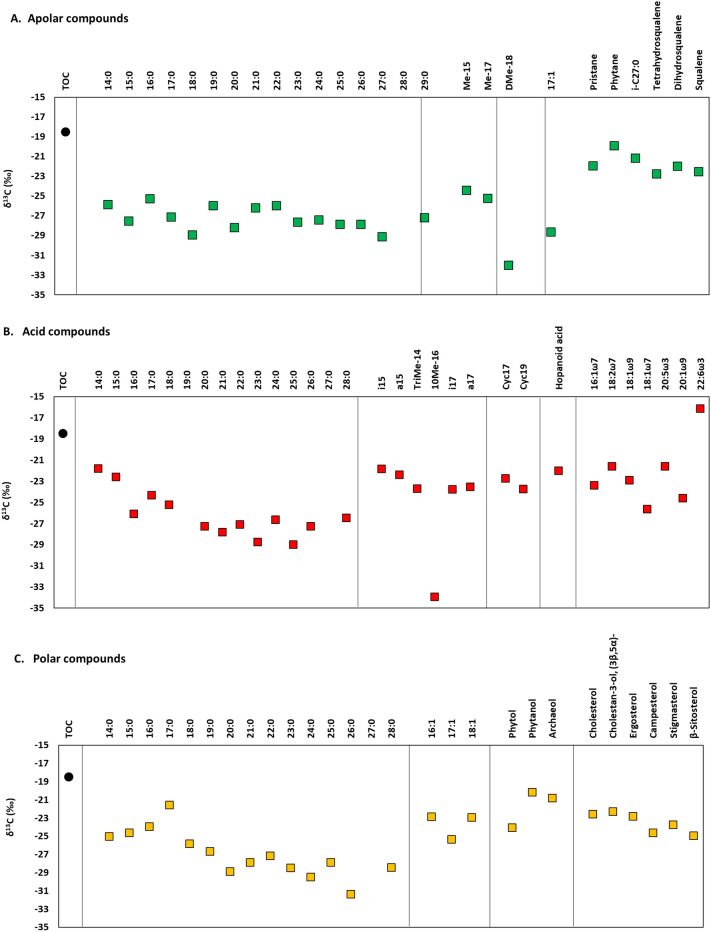
Compound-specific isotopic composition (δ^13^C) of the lipid compounds identified in Tirez in analyses carried out in 2021, in the three polarity fractions; (**A**) apolar, (**B**) acid, and (**C**) polar. The isotopic composition of the total biomass (δ^13^C_TOC_) is shown in each fraction for comparison.

The compound-specific isotopic analysis of the lipids yielded δ^13^C values ranging from − 32.0 to − 19.9‰ among the hydrocarbons (apolar fraction), from − 33.9 to − 16.1‰ among the alkanoic acids (acid fraction), and from − 31.3 to − 20.1‰ among the alcohols (polar fraction). Individual δ^13^C values for biomarkers of a common source in the apolar fraction were: − 27.1‰ for *n*-C_17_, − 28.6‰ for C_17:1_, or ~ − 25‰ for 7Me-C_15_ and 7Me-C_17_ (likely from cyanobacteria); or − 22.5‰ for squalene, − 22.0‰ for dihydrosqualene, and − 22.8‰ for tetrahydrosqualene (most likely from haloarchaea) (Fig. 4A). In the acid fraction, markers of mostly bacterial such as the *n*-C_16:0_ or an undefined hopanoid acid showed δ^13^C values of − 26.0‰ and − 22.0‰, respectively; the gram-positive bacterial C_18:1[ω9]_ had a value of − 22.9‰; the gram positive or SRB-related *iso*/*anteiso* pairs of C_15:0_ and C_17:0_ showed ranges from − 18.5 to − 17.6‰: Cy_17_ of − 22.7‰; and the mid-chain10MeC_16:0_ had a δ^13^C of − 33.9‰ (Fig. 4B). In the polar fraction, the archaeal biomarker archaeol showed a δ^13^C of − 20.7‰; the eukaryotic cholesterol and phytosterols of − 24.9 to − 22.3‰; and the phototrophic phytol of − 24.0 (Fig. 4C).

## Discussion

### The ecological baseline in Tirez

The geology and the climate of the Tirez region favored the generation and maintenance of a type of hypersaline habitat characterized by extreme seasonality: the sulfate-chlorine waters, with sodium and magnesium cations, showed significant seasonal variations^15^. The alkaline pH, the low oxidant value for the redox potential of the water column and the highly reduced sediments imposed extreme conditions (see Table 1 and Supplementary Information for details). This extreme seasonality requires to define a valid representative ecological baseline to compare the ecology of the lagoon between 2002 and 2021 and, in this way, set the basis to proposing our model of ecological succession with increasing dryness as a “time-analog” for early Mars. Taxonomic data from 2002 is a snapshot of the community during one season, so we include in our discussion the results presented by Montoya et al. (2013) from a sample campaign carried out in 2005, because they^16^ analyzed both water and sediment and during both the wet and dry seasons.

We consider here only the results obtained by Montoya et al.^16^ by gene cloning, since those obtained by isolation and sequencing are not comparable. At the level of large groups, no major seasonal differences were observed: Pseudomonadota, followed by Bacteroidetes, were the dominant phyla, in both water and sediments, and both in the dry and the rainy seasons; although Alphaproteobacteria was the dominant class in water, while Gammaproteobacteria was dominant in sediments (in both dry and rainy seasons). With respect to the archaeal domain, all the identified sequences were affiliated to Halobacteriales order, mainly *Halorubrum* (water) and *Halobacterium* (sediment), both within Halobacteriaceae family. We can consider these results presented in Montoya et al.^16^ as the “ecological baseline” for Tirez, however taken with a grain of salt, because only 43 bacterial and 35 archaeal sequences, including rainy and dry seasons and water and sediments, were considered for analysis.

### Prokaryotic diversity in 2002

As can be expected for an extreme environment, the bacterial diversity detected in 2002 was low, although we cannot exclude the possibility that this may reflect the limitation of DNA sequencing techniques at the time. 59% of the obtained clones in the then-wet sediments corresponded to the *Malaciobacter* genus. *Malaciobacter* (previously named^17^ *Arcobacter*) is an aerotolerant Epsilonproteobacteria. Species within this genus are moderately halophilic, e.g., *M. halophilus*, capable to grow in up to 4% NaCl. Even though the role that *Malaciobacter* can play in the environment is not known, it seems to thrive in aquatic systems, like sewage, with a high organic matter content^17^: e.g., *M. canalis*, *M. cloacae*, or *M. defluvii*.

After Malaciobacter-like clones*,* the next most numerous group belongs to the phylum Bacillota (27% of the sequenced clones; Table 2). Under stressful environmental conditions, members of the genus *Virgibacillus* produce endospores, a very useful property in an extreme and variable environment (ionic strength, temperature, light intensity), easy to compare with early Mars. Endospores facilitate species survival, allowing them to overcome drastic negative changes, like dry periods, and to germinate when the conditions are favorable again. The closest identified species was the halotolerant *V. halodenitrificans*, but with low homology, not far from other halotolerant (e.g., *V. dokdonensis*) or halophilic (e.g., *V. marismortui*) species within the same genus. The other Gram-positive clones belong to the order Clostridiales. These clones cluster in two taxonomic units related with the strictly anaerobic genus *Tissierella*.

Despite the abundance of Pseudomonadota, their biodiversity was very low, reduced to only two genera within the Epsilon- and Delta-proteobacteria. Six sequences affiliated to Deltaproteobacteria, and clustered in one OTU (salB38, similarity 96.6% with *Desulfotignum*), were retrieved. Its presence in anaerobic media rich in sulfates (Table 1) seems reasonable. In fact, sulfate-reducing activity was detected using a specific enrichment assay.

Finally, one taxon belonging to the phylum Spirochaetota (previously named Spirochaetes) was identified. The presence of Spirochaetota in this system is not strange because members of the genus *Spirochaeta* are very often found in mud and anaerobic marine environments rich in sulfates^18^. Moreover, the closest species to SalB63, although with a low similarity of 87%, was *Spirochaeta bajacaliforniensis*, a spirochete isolated^19^ from a microbial mat in Laguna Figueroa (Baja California), an extensive hypersaline lagoon with high gypsum content, very similar, although much bigger, than Tirez lagoon.

The diversity within the domain Archaea was very low in 2002. The phylogenetic analysis of 96 clones indicate that they correspond to one specie belonging to the obligate halophile genus *Methanohalophilus*. Their high similarity (99.3%) with several species of *Methanohalophilus*, such as *M. portucalensis* (isolated from sediments of a solar saltern in Portugal), *M. mahii* (isolated from sediments of the Great Salt Lake), or *M. halophilus* (isolated^20^ from a cyanobacterial mat at Hamelin Pool, Australia), makes impossible its adscription to any particular species level. *Methanohalophilus* is strictly methylotrophic, which is consistent with this environment, given that the methylotrophic methanogenesis pathway, non-competitive at low-salt conditions, is predominant at high saline concentrations^21^. We further confirmed methanogenic activity in Tirez by the measurement of methane by gas chromatography in enrichment cultures.

### Prokaryotic diversity in context of other studies between 2002 and 2021

It was challenging to establish a timeline for the succession of the populations involved, because the scarcity of data harvested and published so far from Tirez. However, combining our results with the few data available in Montoya et al.^16^ and Preston et al.^22^ on samplings carried out on 2005 and 2017, respectively, we can see a clear predominance of the phylum Pseudomonadota: Epsilonproteobacteria, i.e. Arcobacter-like, and Deltaproteobacteria, mainly sulfate-reducing bacteria (this work, sampling 2002), and Gammaproteobacteria^16^ when Tirez maintained a water film, to eventually a final predominance of Gammaproteobacteria, e.g. Chromatiales and Pseudomonadales, in the dry Tirez (this work, 2020 sampling). The Bacillales order has remained widely represented both in the wet and dry Tirez.

Regarding the archaeal domain, the few references available (Refs.^16,22^; this work) confirm that the members of the Halobacteriaceae family are well adapted to both the humid and dry ecosystems of Tirez, being predominant in both conditions. Preston et al.^22^ found that the second most abundant group of archaea in the dry sediments of Tirez was the Methermicoccaceae family, within the Methanosarcinales order, Methanomicrobia class. Taking into account the results obtained in the dry Tirez (Preston et al.^22^; and this work, sample 2020), the methanogenic archaea have decreased drastically through time, probably due to salt stress and the competition with sulfate-reducing bacteria.

### Prokaryotic diversity in 2021

From a metabolic point of view, most of the bacteria present today in the sediment are chemoorganotrophs, anaerobes, and halophilic or halotolerant. Scarce information is available about the predominant OTU, Candidate Division OP1. The OP1 division was one of the main bacterial phyla in a sulfur-rich sample in the deepest analyzed samples from the Red Sea sediments under brine pools^23^. In addition, the phylogenetically related Candidate division KB1 has been observed in deep-sea hypersaline anoxic basins at Orca Basin (Gulf of Mexico), and other hypersaline environments^24^. Eight of the nine genera identified show coverage greater than 1% of the sequences: i.e., *Rubinisphaera*, *Halothiobacillus*, *Thiohalophilus*, *Anaerobacillus*/*Halolactibacillus*, *Halomonas*, *Halothermothrix*, and *Aliifodinibius* are halophilic or halotolerant genera^13,25^.

Regarding archaea, our analyses reveal archaeal groups that seem to thrive in sediments from extreme environments, e.g., marine brine pools/deep water anoxic basins or hypersaline lakes. The most abundant OTU, Thermoplasmata KTK4A, was found prominent and active in the sediment of Lake Strawbridge, a hypersaline lake in Western Australia^26^, and in soda-saline lakes in China^27^. The creation of a Candidatus Haloplasmatales, a novel order to include KTK4A-related Thermoplasmata, has been proposed^27^. On the other hand, both in the aforementioned soda-saline lakes in China^27^ and in a sulfur-rich section of the sediments from below the Red Sea brine pools^23^, retrieved sequences were assigned to Marine Benthic Groups B, D, and E. Finally, in the section of nitrogen-rich sediments from the aforementioned Red Sea brine pools, the unclassified lineage ST-12K10A represented the most abundant archaeal group. In the Tirez Lagoon sediment after desiccation, all Methanomicrobia readings belonged to this group.

The significance and implications of an ecosystem characterized in 2021 by high diversity, high inequality, and lack of isolated representatives, resides in that Tirez is today an ecosystem in which many (most) of the species/OTUs present are dormant, and they do not play any metabolic role. Hence the high percentage of raretons, greater than 80% for both bacteria and archaea, which are actually present in the lagoon but with only one or two copies each. Only those species adapted to the conditions imposed by the extreme environment are able to actually thrive, and consequently only a few species carry out all the metabolic activity. We conclude that the microbiota in Tirez today represents an ecosystem with a high resilience capacity in the face of environmental changes that may occur.

We want to clearly highlight that the technique available in 2002 to study the microbiota of the Tirez lagoon only allowed to obtain a low-resolution image, but that was the state-of-the-art procedure at the time, and the Tirez lagoon cannot be sampled again with the conditions back in 2002, which no longer exist and are not expected to return. Although we have kept in storage several samples of water and sediment from the 2002 Tirez lagoon, it is reasonable to assume that those laboratory microcosms would have chemically and microbiologically changed during the last 20 years, and as such no longer represent reliable replicas of the original lagoon, so we cannot use them for the purposes of this work. Therefore, we are aware that any comparisons of the 2002 laboratory results with the much more robust results obtained by Illumina in 2021 need to be taken with a grain of salt. With all the precautions required, in a high-level, first-order comparison, the most noticeable difference between 2002 and 2021 is a drastic change in the microbial Tirez population. Only some OTUs within Bacillales (*Virgibacillus*/*Anaerobacillus*), sulfate-reducing Deltaproteobacteria (*Desulfotignum*/Desulfobacteraceae-*Desulfovibrio*), and Spirochaetes are shared among the 2002 and 2021 samples. This comparison is enough for the purposes of this work, as we are interested in the evolution of the lagoon system as a whole to establish a “time-analog” with the wet-to-dry transition on early Mars, and not in the particular outcome of each and every OTU in Tirez. With the results at hand, we conclude that, since 2002, the lacustrine microbiota has shifted to one more adapted to the extreme conditions in the dry sediments, derived from the gradual and persistent desiccation concluding ca. 7 years ago (i.e., completely desiccated in 2015), such as lack of light, absence of oxygen, and lack of water availability. This shift has likely been triggered because organisms that were originally in the lagoon but at low abundance in 2002 became dominant as they were better adapted to desiccation, and because the incoming of new microorganisms transported by birds or wind^28^.

### Lipid biomarkers analysis of the desiccated lake sediments

The analysis of cell membrane-derived lipid compounds on the dry lake sediments at present allow to provide another perspective of the microbial communities inhabiting the Tirez lagoon, by contributing additional information about the ecosystem and depositional environment. It is important to note that, analyzing only the 2021 lake sediments, we cannot differentiate between lipidic biomarkers of the microorganisms inhabiting Tirez in 2002 and before from those left behind by the microorganisms living in the dried sediments today. Instead, the analyses of lipid biomarkers provide clues about the different microorganisms that have populated Tirez through time, including both older communities inhabiting the former aqueous system and also younger communities better adapted to the present dry conditions. Thus, the lipid biomarkers analysis can be considered as a time-integrative record of the microbial community inhabiting Tirez during the last decades.

Based on the molecular distribution of lipid biomarkers, the presence of gram-positive bacteria was inferred from the relative abundance of the monounsaturated alkanoic acid C_18:1[ω9]_, or *iso/anteiso* pairs of alkanoic acids from 12 to 17 carbons^29^ with dominance of *i*/*a*-C_15:0_ and *i*/*a*-C_17:0_ (Fig. 3B). In contrast, generally ubiquitous alkanoic acids such as C_16:1[ω7]_, C_18:1[ω7]_, or C_18:2[ω6]_ suggested a provenance rather related to gram-negative bacteria^30^. The combined detection of the *i*/*a*-C_15:0_ and *i*/*a*-C_17:0_ acids, with dominance of the *iso* over the *anteiso* congeners, together with other biomarkers such as the mid-chain branched 10Me16:0, the monounsaturated C_17:1_, or the cyclopropyl Cy17:0 and Cy19:0 acids, may be associated with a community of SRB^31^ in today´s dry sediments of Tirez. Specifically, most of those alkanoic acids have been found in a variety of Deltaproteobacteria and/or Bacteroidota (previously named Bacteroidetes). The presence of archaea was deduced from the detection of prominent peaks of archaeol in the polar fraction^32^ (Fig. 3C), as well as squalene and relatives (dihydrosqualene and tetrahydrosqualene) in the apolar fraction^33^ (Fig. 3A). Squalene and a variety of unsaturated derivatives are present in the neutral lipid fractions of many archaea with high abundances in saline lakes^34^. The relative abundance of autotrophs over heterotrophs^35^ can be estimated by the ratio of the autotrophically-related pristane and phytane over the both autotrophically- and heterotrophically-produced *n*-C_17_ and *n*-C_18_ alkanes ([Pr + Ph]/[*n*-C_17_ + *n-*C_18_]). A ratio of 0.56 in the Tirez sediments suggest the presence of a relevant proportion of heterotrophs in the ancient lacustrine system.

Furthermore, the lipid biomarkers analysis was able to detect compounds specific of additional microbial sources, such as cyanobacteria^36^ (*n*-C_17_, C_17:1_, or 7Me-C_15_ and 7Me-C_17_), microalgae and/or diatoms (phytosterols^37^; or C_20:5_, and C_22:6_ alkanoic acids^30^), and other photoautotrophs (phytol and potentially degradative compounds such as pristane and phytane^31^). A relatively higher preservation of the cell-membrane remnants (i.e., lipids) compared to the DNA-composing nucleic acids may contribute to explain the lack of detection of cyanobacteria, diatoms and microalgae, and other phototrophs by DNA analysis (a deficit in our results shared with Montoya et al.^16^, and Preston et al.^22^). Although abundant in higher plants^38^, sterols such as those detected here (i.e., the sterols campesterol, stigmasterol, and β-sitosterol, as well as ergosterol) are also major sterols in some microalgal classes^37^ (such as Bacillariophyceae, Chrysophyceae, Euglenophyceae, Eustigmatophyceae, Raphidophyceae, Xanthophyceae, and Chlorophyceae), cyanobacteria (β-sitosterol), and fungi (ergosterol^39^).

The carbon isotopic composition of lipid biomarkers provides a rapid screening of the carbon metabolism in a system, by recognizing the principal carbon fixation pathways used by autotrophs. The range of δ^13^C values measured in the Tirez sediments (from − 33.9 to − 16.1‰) denotes a mixed use of different carbon assimilation pathways, involving mostly the reductive pentose phosphate (a.k.a. Calvin–Benson–Bassham or just Calvin) cycle (from − 19 to − 30‰), and in lesser extent the reductive acetyl-CoA (a.k.a. Wood–Ljungdahl) pathway (from − 28 to − 44‰), and/or the reverse tricarboxylic acid (rTCA) cycle (from − 12 to − 21‰).

The lipids synthesized by microorganisms using the Calvin or reductive acetyl-CoA pathway are typically depleted relative to the bulk biomass, particularly those produced via de latter pathway. In the dry Tirez sediments, the majority of the lipid compounds are more depleted in ^13^C than the bulk biomass (Fig. 4). In particular, the branched alkane DiMeC_18_ (Fig. 4A) and the SRB-indicative 10Me16:0 acid (Fig. 4B) showed the most depleted δ^13^C values and suggested the use of the reductive acetyl-CoA pathway. The rest of lipid compounds showed isotopic signatures (from − 16.1 to − 31.4‰) compatible with the prevalence of the Calvin pathway. These values may directly reflect the autotrophic activity of microorganisms fixing carbon via the Calvin cycle or heterotrophic activity of microorganisms growing on their remnants. Thus, the saturated and linear alkyl chains of lipids (i.e., *n*-alkanes, *n*-alkanoic acids, and *n*-alkanols) showing the most negative δ^13^C values (e.g., alkanes *n*-C_17_ and C_17:1_; or acid C_18:1[ω7]_) reflect prokaryotic sources of Calvin-users autotrophs (e.g., cyanobacteria or purple sulfur bacteria), while the rest of compounds with slightly less negative δ^13^C values instead stem from the autotrophic activity of eukaryotes also users of the Calvin cycle (unsaturated fatty acids and sterols) or from the metabolism of heterotrophs such as SRB (*iso/anteiso*-, other branched, and cyclopropyl fatty acids) and haloarchaea (isoprenoids, phytanol, and archaeol). All in all, the compound-specific isotope composition of the dry sediments in the today´s Tirez lagoon may indirectly reflect the prevailing autotrophic mechanisms in the present lacustrine system of Tirez, by showing isotopic signatures of secondary lipids similar to their carbon source^40^.

In addition, the use of a number of lipid molecular ratios or proxies allow further characterization of the lacustrine ecosystem and depositional environment. For example, the average chain length of the *n*-alkanes (24.1) suggests a relevant presence of eukaryotic biomass in the lacustrine sediments, since long-chained alkanes (> C_20_) are known to originate from epicuticular leaf waxes in higher plants^41^. Highlighting the relevance of eukaryotes and their ecological roles is one of the major contributions of this work, because previous studies on the microbial ecology of hypersaline environments have been focused primarily on prokaryotes^42^.

The proportion of odd n-alkanes of high molecular chain (i.e., *n*-C_27_, *n*-C_29_, and *n*-C_31_) over even *n*-alkanes of low molecular chain (i.e., *n*-C_15_, *n*-C_17_, and *n*-C_19_) provides an estimate of the relative abundance of terrigenous over aqueous biomass^43^, which in Tirez is TAR = 1.8. The P_aq_ index may also be used to differentiate the proportion of terrigenous versus aquatic (emergent and submerged) plant biomass^44^. A P_aq_ of 0.3 in the Tirez sediments from 2021 supported the relative abundance of land plants. Finally, the depositional environment in the lacustrine system of Tirez may be also characterized analyzing the ratio of pristane over phytane (Pr/Ph), which is higher than 1 when phytol degrades to pristane under oxic conditions^45^. Assuming that both isoprenoids in the Tirez sediments derived from phytol^31^, according to their similarly depleted δ^13^C (Fig. 4A), we can conclude that the sediments in the Tirez lagoon were deposited under predominantly oxic conditions (i.e., Pr/Ph ratio of 1.1).

In summary, the lipid biomarkers study revealed useful information about the depositional environment and lacustrine ecosystem, including the presence of active or past autotrophic metabolisms involving prokaryotes (e.g., cyanobacteria and purple sulfur bacteria) and eukaryotes (plants, diatoms and other microalgae), as well as heterotrophic metabolisms of likely SRB and haloarchaea growing on Calvin-users exudates. These results are quite in agreement with the microbial community previously reported^16^ in sediments from the wet and dry seasons: abundant Gammaproteobacteria and Alphaproteobacteria, together with Algae and Cyanobacteria, dinoflagellates and filamentous fungi, Bacillota, Actinomycetota (previously named Actinomycetes), and a halophilic sulfate-reducing Deltaproteobacteria.

### Tirez as the first astrobiological “time-analog” for early Mars

Early Mars most likely had a diversity of environments in terms of pH, redox conditions, geochemistry, temperature, and so on. Field research in terrestrial analog environments contribute to understand the habitability of this diversity of environments on Mars in the past, because terrestrial analogues are places on Earth characterized by environmental, mineralogical, geomorphological, or geochemical conditions similar to those observed on present or past Mars^9^. Therefore, so far analogs have been referred to terrestrial locations closely similar to any of the geochemical environments that have been inferred on Mars, i.e., they are “site-analogs” that represent snapshots in time: one specific environmental condition at a very specific place and a very specific time. Because of this, each individual field analog site cannot be considered an adequate representation of the changing martian environmental conditions through time. Here we introduce the concept of astrobiological “time-analog”, referred to terrestrial analogs that may help understand environmental transitions and the related possible ecological successions on early Mars. In this sense, they should be “time-resolved analogs”: dynamic analog environments where we can analyze changes over time. To the best of our knowledge, this is the first study that looks at the environmental microbiology of a Mars astrobiological analog site over a significant and long period of change, and try to understand the ecological successions to put them in the context of martian environmental evolution.

As Mars lost most of its surface water at the end of the Hesperian^5,9,12^, this wet-to-dry global transition can be considered the major environmental perturbation in the geological history of Mars, and therefore merits to be the first one to be assigned a “time-analog” for its better understanding and characterization. The drying of Mars was probably a stepwise process, characterized by multiple transitions between drier and wetter environments^12,47^, and therefore the seasonal fluctuations and eventual full desiccation of Tirez represent a suitable analog to better understand possible ecological transitions during the global desiccation of most of the Mars’s surface before the Amazonian (beginning 3.2 Ga).

To introduce Tirez as the first Mars astrobiological “time-analog” of the wet-to-dry transition on early Mars, the objective of this study was threefold: first, we wanted to identify the dominant prokaryotic microorganisms in the active Tirez lagoon 20 years ago, a unique hypersaline ecosystem with an ionic composition different from that of marine environments, and therefore potentially analogous to ancient saline lacustrine environments on Mars during the Noachian and into the Hesperian^46,47^. Our results provide a preliminary basis to hypothesize how the microbial communities on the Noachian Mars could have developed in salty environments with dramatically fluctuating water availability. The requirement to deal with important variations in ionic strength and water availability, involving at times the complete evaporation of the water, could have represented additional constraints^48^ for microorganisms on early Mars.

The second objective of this investigation was the identification of the microbial community inhabiting the desiccated Tirez sediments today, after all the water was lost, as a potential analog to desiccated basins on Mars at the end of the Hesperian^1,3,4,47^. Our results suggest that hypothetical early microbial communities on early Mars, living with relative abundance of liquid water during the Noachian, would have been forced to adapt to increasingly desiccating surface environments, characterized by extreme conditions derived from the persistent dryness and lack of water availability. Our investigation in Tirez suggest that hypothetical microorganisms at the end of the Hesperian would have needed to evolve strategies similar to those of microorganisms on Earth adapted to living at very low water activity^49^, to thrive in the progressively desiccating sediments.

And the third objective of this investigation was the identification of the lipidic biomarkers left behind by the microbial communities in Tirez, as a guide to searching and identifying the potential leftovers of a hypothetical ancient biosphere on Mars. Lipids (i.e., fatty acids and other biosynthesized hydrocarbons) are structural components of cell membranes bearing recognized higher resistance to degradation relative to other biomolecules, thus with potential to reconstruct paleobiology in a broader temporal scale than more labile molecules^50^. Our results reinforce the notion that lipidic biomarkers should be preferred targets in the search for extinct and/or extant life on Mars precisely because they are so recalcitrant.

## Conclusions

The Tirez lagoon, when still active ca. 20 years ago, was a model of extreme halophilic environment. The microorganisms in Tirez have had to adapt not only to high concentration of salts, but also to the strong cyclic seasonal changes. As can be expected for an extreme environment, the bacterial diversity detected was low (although we do not exclude the possibility that this may reflect the limitation of DNA sequencing techniques at the time), and the archaeal diversity was reduced to only one taxon belonging to a halophilic methylotrophic methanogen. Perhaps the most outstanding property of the prokaryotic communities in the former lagoon in Tirez was their ability to deal with important seasonal changes (ionic strength, water, oxygen), which in some cases involved the complete evaporation of the water during the summer. After several years of complete desiccation (in ca. 2015), the microbiota has shifted to become mostly related to clusters without isolated members, in some cases groups detected in unusual environments such as deep-sea hypersaline anoxic basins. The molecular and isotopic fingerprints of lipids biomarkers left behind by the microbial communities inhabiting Tirez through time inform about research strategies to search for possible biomarkers left behind by such communities after all the water was lost.

All these characteristics make Tirez a suitable analog for the changing paleoenvironmental conditions on Mars during the Late Hesperian to the Early Amazonian, as revealed by spacecraft investigations. Several identified paleolakes on Mars were characterized by episodic inundation by shallow surface waters with varying salinity, evaporation, and full desiccation repeatedly over time, until the final disappearance of most surface water after the wet-to-dry transition at the end of the Hesperian. We have described here that similar conditions can be tested through time in the terrestrial analog Tirez lagoon, including ecological successions, and we have demonstrated that the lagoon was habitable for a wide range of prokaryotes before and after its complete desiccation, in spite of the repeated seasonal dryness. Our 25 yearlong analyses of the geobiological transitions in the Tirez lagoon represent the first terrestrial astrobiological “time-analog” for desiccating saline lakes on early Mars.

## Methods

### Characterization of the active lake sediments in 2002

#### Sampling and physico-chemical characterization

The sampling was done the first days of April 2002, with average temperature and rain values of 13 °C and 45 mm, respectively. Table 1 describes the physico-chemical parameters in Tirez from samples taken at the end of the then-wet season. Sampling was performed with a RingKit core-sampler for soft soils. A homogeneous mixture of the first 6 cm of sediments with the water retained in the core-sampler, corresponding to a 1–2 cm of the water column, was taken.

#### Metabolic assays

Sulfate-reducing and methanogenic activities were analyzed using the mixture of sediment in water as inoculum. For enrichment cultures of sulfate-reducing bacteria (SRB) the following media was used^40^: 0.2% MgSO_4_·7H_2_O, 0.35% Na-lactate, 0.1% Fe(NH_4_)_2_·6H_2_O, 1 ml/l of trace elements, in Tirez lagoon water. The sulfide production was qualitatively detected with the lead acetate-paper method. The methanogenic activity assay was carried out as described previously^51^. Acetate and formate were used as substrates. The methane produced was analyzed with a Shimadzu GC-8A gas chromatograph.

#### DNA extraction, clone libraries, and sequence analysis

Cells from the homogeneous sediment–water samples were disrupted and DNA was extracted using FastDNA kit for soils BIO101 according to the manufacturer’s protocol. To obtain 16S rRNA genes two oligonucleotide primer pairs were used: 27F and 1492R (annealing T: 56 °C) for the Bacteria domain^52^, and 25F and 1492R (annealing T: 52 °C) for the Archaea domain^52^. The amplicons were cloned using TOPO Cloning Kit (Invitrogen Corporation, San Diego, California) and then transformed into competent *E. coli* cells. Plasmid DNA inserts were extracted by alkaline lysis method (Miniprep^53^). Plasmid inserts were amplified by PCR using the M13 primer set (Invitrogen). Automated DNA sequencing was performed with an ABI model 377 sequencer (Applied Biosystems).

Sequences were compared with the NCBI database by using the basic local alignment search tool (BLAST, http://www.ncbi.nim.gov) to identify the closest sequence. The sequences obtained in this study were deposited in the GenBank database under accession numbers: EF031086-EF031097**.**

### Characterization of the dried lake sediments in 2021

#### Sampling

The samples were collected in June 2021, with the lagoon completely dry. Four samples, from a black band of sediment located at a depth of 5–10 cm, were sampled and pooled for further analysis. The pH and redox potential were 7.3 and − 11 mV, respectively.

#### DNA extraction and Illumina sequencing, and Phylogenetic analysis

Total DNA was extracted using the FastDNA^®^ SPIN Kit for soil and FastPrep^®^ Instrument (MP Biomedicals, Santa Ana, USA). Massive sequencing was carried out using the primer sets PRO345F (CCTACGGGNBGCASCAG)/PRO 805R (GACTACNVGGGTATCTAATCC) for the Bacteria Domain^54^, and A340F (CCCTAYGGGGYGCASCAG)/A806R (GGACTACVSGGGTATCTAAT) for the Archaea Domain^55^, and performed by a MySEq V3 (2X300pb) platform (Illumina, San Diego, USA) at FISABIO Sequencing and Bioinformatics Service (Valencia, Spain).

The sequence processing was performed using the Mothur^56^ package v.1.36.0 (http://www.mothur.org). Any sequences with low quality base scores (i.e., Phred quality scores < 25) were removed. Sequencing noise was removed by the Pre.cluster tool in the Mothur package and Chimeras introduced in the PCR process were detected and removed using ChimeraUquime. Qualified sequences were then clustered into operational taxonomic units (OTUs) defined by a 3% distance level based on the distance matrix and a bootstrap higher than 60%. Taxonomic classification was performed using the SILVA 16S rRNA gene database, using a k-nearest neighbor consensus and the Wang approach. Confidence values less than 80% at a phylum level, were considered unclassified^57^. Additional statistical and graphical analyses were conducted with the package Vegan^58^ for program R (http://www.R-project.org/).

The data obtained in this study were registered with the National Center for Biotechnology Information (NCBI) under the BioProject identifier PRJNA849409. The data set containing the sequence reads was deposited in the BioSamples database, accessible under the ID numbers SRX15710472 for Bacteria and SRX15710473 for Archaea.

### Characterization of lipid biomarkers in 2021

#### Lipid biomarkers extraction and analysis

A lyophilized and ground sample (17 g) of subsurface sediments (5–10 cm) from the dry lake was extracted with ultrasound sonication following the method described elsewhere^59^.

The concentrated and desulfurized total lipids extract^48^ was hydrolyzed overnight with KOH (6% MeOH) at room temperature^60^. Then, a liquid–liquid extraction^61^ with *n*-hexane was performed to recover the neutral fraction after acidification with HCl (37%). Further separation of the neutral fraction into apolar (hydrocarbons) and polar (alkanols and sterols) fractions was done according to a method described in detail elsewhere^62^. The acidic fraction was recovered with *n*-hexane from the hydrolyzed and acidified lipid extract and transesterified with BF_3_ in MeOH to produce fatty acid methyl esters (FAMEs). The polar fraction was trimethylsilylated (N,O-bis [tri- methylsilyl] trifluoroacetamide [BSTFA]) to analyze the resulting trimethyl silyl alkanols^61^. The three fractions (containing the hydrocarbons, trimethylsilylated alcohols, or methylated alkanoic acids) were analyzed using gas chromatography-mass spectrometry (GC–MS) by a 6850 GC System coupled to a 5975C VL MSD Triple-Axis detector (Agilent Technologies, Santa Clara, CA, USA) operating with electron ionization at 70 eV and scanning from *m/z* 50 to 650 (analytical details can be found in Ref.^61^). Compound identification was based on retention time and mass spectra comparison with reference materials and the NIST mass spectral database. Quantification was performed with the use of external calibration curves of *n*-alkanes (C_10_ to C_40_), FAMEs (C_6_ to C_24_) and *n*-alkanols (C_14_, C_18_ and C_22_), all supplied by Sigma-Aldrich (Madrid, Spain). The recovery of three internal standards was measured to average 75 ± 17%.

#### Bulk geochemistry and compound-specific isotope analysis of lipid biomarkers

The stable isotope composition of organic carbon (δ^13^C) and total nitrogen (δ^15^N) was measured on the bulk sample using isotope-ratio mass spectrometry (IRMS), following the USGS method^63^. The carbon and nitrogen isotopic composition of the sample was measured in a MAT 253 IRMS (Thermo Fisher Scientific, Waltham, Massachusetts, USA) and the resulting δ^13^C and δ^15^N values were reported in the standard per mil notation (‰). Three certified standards were used (USGS41, IAEA-600 and USGS40) with an analytical precision of 0.1‰. The content of total nitrogen (TN %) and total organic carbon (TOC %) was measured with an elemental analyzer (HT Flash, Thermo Fisher Scientific, Waltham Massachusetts, USA) during measurement of the stable isotopes.

The carbon isotopic composition of individual lipid compounds was determined for the three polarity fractions by coupling the gas chromatographer-mass spectrometer (Trace GC 1310 ultra and ISQ QD-MS) to the isotope-ratio mass spectrometry system (MAT 253 IRMS, Thermo Fisher Scientific). Details on the GC-IRMS conditions, sample injection and analysis, as well as isotopic composition calculations are provided elsewhere^62^. For the alkanoic acids, the δ^13^C data were calculated from the FAME values, correcting them for the one carbon atom added in the methanolysis^64^. For *n*-alkanols and other polar compounds, the δ^13^C data were calculated from the trimethylsilylated derivate, correcting them for the three carbon atoms added in the derivatization process with BSTFA. All δ^13^C values are reported relative to the Pee Dee Belemnite (PDB) standard.

## Supplementary Information

Supplementary Information.

## Data Availability

The sequences obtained in this study were deposited in the GenBank database under accession numbers EF031086-EF031097, and in the BioSamples database under the ID numbers SRX15710472 and SRX15710473. Other datasets generated during and/or analysed during the current study are available from the corresponding author on reasonable request.
