# Supplementary material for: Ecological successions throughout the desiccation of Tirez lagoon (Spain) as an astrobiological time-analog for wet-to-dry transitions on Mars
Source: Sci Rep. 2023 Feb 8;13:1423. doi: 10.1038/s41598-023-28327-3 (PMC9908944; doi:10.1038/s41598-023-28327-3)
Supplement: Supplementary file 1 — Supplementary Information. [file 41598_2023_28327_MOESM1_ESM.docx]

**Supplementary Information for**

**Ecological successions throughout desiccation of Tirez lagoon (Spain) as an astrobiological time-analog for wet-to-dry transitions on Mars**

Alberto G. Fairén, Nuria Rodríguez, Laura Sánchez-García, Patricia Rojas, Esther R. Uceda, Daniel Carrizo, Ricardo Amils, José L. Sanz

From a limnological point of view, Tirez was a rather peculiar lagoon, with a differential precipitation of various types of salts and crust formations during the summer. The salt content of the sulfate-chlorine magnesium-sodium waters in Tirez varied between 100 and 250 g/l, and significant oscillations in water content, temperature, pH and dissolved oxygen were measured depending on the season. Due to the high sulfate and magnesium content in the water, it was classified as a Na-Mg-Cl-SO_4_ type of lagoon^15^. During the dry season, the inner part of the lagoon was covered by a saline crust made up of (from top to bottom) halite, epsomite, thenardite, bloedite and gypsum. From a lithological point of view, the dominant material was sand, which alternates with gypsum and limestone^65^. The bottom was made of clay with a sapropel layer produced by the decomposition of algae with epsomite crystals^15^.

Information on microorganisms from athalassohaline environments, such as Tirez, which are normally small, shallow, endorheic and highly seasonal continental lagoons, are scarce. The extreme conditions in which life must develop in these systems, including high variability of salinity in a short period of time, temperature, light intensity, and others, impose strict restrictions to their colonization. There are some reports on planktonic organisms in these peculiar water bodies^66,67^, although microbial mats, which can be the dominant biota of these systems, have been more extensively studied^68,69^. Previous studies are in general descriptive reports emphasizing the presence of phototrophic oxygen-producing microorganisms, such as *Oscillatoria* spp., *Gleocapsa* spp. and *Microcoleus chthonoplastes*, the latter cited as the dominant species in the microbial mats from La Mancha lagoons^70^.

**Supplementary References**

65. Prieto-Ballesteros, O., Rodríguez, N., Kargel, J.S., Kessler, C.G., Amils, R., and Remolar, D.F. (2003) Tirez Lake as a terrestrial analog of Europa. Astrobiology 3:863–877.

66. Jiang H., Dong H., Zhang G., Yu B., Chapman L.R., Fields M.W. 2006. Microbial diversity in water and sediment of Lake Chaka, an athalassohaline lake in northwestern China. Appl Environ Microbiol. 72(6): 3832-3845. doi: 10.1128/AEM.02869-05

67. Thombre R. S., Gomez F., Parkhe R., Kaur K., Vaishampayan P., Shivakarthik E., Sivaraman B., Perumal R., Mason N. 2020. Effect of impact shock on extremophilic Halomonas gomseoemensis EP-3 isolated from hypersaline sulphated lake Laguna de Peña Hueca, Spain. PSS. 192: 105041. doi: 10.1016/j.pss.2020.105041

68. Mouné, S., Caumette, P., Matheron, R., and Willison, J. C. (2003). Molecular sequence analysis of prokaryotic diversity in the anoxic sediments underlying cyanobacterial mats of two hypersaline ponds inMediterranean salterns. FEMSMicrobiol. Ecol. 44, 117–130. doi: 10.1016/S0168-6496(03)00017-5F

69. Farías, M.E., Contreras, M., Rasuk, M.C., Kurth, D., Flores, M.R., Poiré, D.G., Novoa, F., Visscher, P.T. (2014) Characterization of bacterial diversity associated with microbial mats, gypsum evaporites and carbonate microbialites in thalassic wetlands: Tebenquiche and La Brava, Salar de Atacama, Chile. Extremophiles 18 (2), 311–329. Doi 10.1007/s00792-013-0617-6

70. Guerrero MC, Tadeo AB, de Wit R (1994) Environmental factors controlling the development of microbial mats in inland saline lakes; the granulometric composition of the sediment In: Stal LJ, Caumette P (ed) Microbial Mats, NATO ASI Series, vol G35, Springer-Verlag, Berlin, pp 85-90
